# Finite Element Analysis of the Mechanism of Traumatic Aortic Rupture (TAR)

**DOI:** 10.1155/2020/6718495

**Published:** 2020-07-07

**Authors:** JiFeng Nan, Mohammadreza Rezaei, Rashid Mazhar, Fadi Jaber, Farayi Musharavati, Erfan Zalnezhad, Muhammad E. H. Chowdhury

**Affiliations:** ^1^Department of Mechanical Engineering, Hanyang University, Seoul, Republic of Korea; ^2^Thoracic Surgery-Pulmonology, Hamd Hospital, Doha, Qatar; ^3^Department of Biomedical Engineering, Ajman University, Ajman 2758, UAE; ^4^Mechanical and Industrial Engineering Department, College of Engineering, Qatar University, P.O. Box 2713, Doha, Qatar; ^5^Department of Biomedical Engineering, UTSA, Texas 78249, USA; ^6^Department of Electrical Engineering, Qatar University, Doha 2713, Qatar

## Abstract

As many as 80% of patients with TAR die on the spot while out of those reaching a hospital, 30% would die within 24 hours. Thus, it is essential to better understand and prevent this injury. The exact mechanics of TAR are unknown. Although most researchers approve it as a common-sense deceleration injury, the exact detailed mechanism of TRA still remains unidentified. In this work, a deceleration mechanism of TAR was carried out using finite element analysis (FEA). The FE analysis aimed to predict internal kinematics of the aorta and assist to comprehend the mechanism of aorta injury. The model contains the heart, lungs, thoracic aorta vessel, and rib cage. High-resolution computerized tomography (HR CT scan) was used to provide pictures that were reconstructed by MIMICS software. ANSYS FE simulation was carried out to investigate the behavior of the aorta in the thoracic interior after deceleration occurred during a car crash. The finite element analysis indicated that maximum stress and strain applied to the aorta were from 5.4819e5 to 2.614e6 Pa and 0.21048 to 0.62676, respectively, in the *Y*-direction when the initial velocity increased from 10 to 25 m/s. Furthermore, in the *X*-direction when the velocity changed from 15 to 25 m/s, the stress and strain values increased from 5.17771e5 to 2.3128e6 and from 0.22445 to 0.618, respectively.

## 1. Introduction

### 1.1. Background

Traffic accident casualties have become a threat to public safety causing major mortality, morbidity, and social losses. One of the most dangerous causes of death in car crashes is blunt aortic injury leading to traumatic aortic rupture (TAR). TAR is presently the second leading cause of death after automobile crashes [1]. Historical records show that 30% of all victims of motor vehicle accidents suffered from a chest injury. Researches from the American Association for Surgery of Trauma indicate that about 8000 victims die due to TAR each year [2, 3]. Overall, TAR is responsible for up to 16% of automobile collision deaths [4]. Of the TAR victims, nearly 80% die on the scene of the accident, while 30% of those reaching hospitals alive, die within the next 24 hours [5]. The aortic isthmus, at the attachment of the arterial ligament which is located on the inner side of the junction of the aortic arch and the descending aorta, is conventionally regarded as the weakest area in the aorta. Researchers from the William Lehman Injury Research Center also pointed out that 94% of all TAR injuries include the isthmic area [6].

TAR incidents from 1998 to 2006 were reviewed by Bertrand et al. [7] utilizing documents from the UK Cooperative Crash Injury. They showed that TAR was the cause for 21.4% of all deaths and that 1.2% of all victims experienced different kinds of TAR. The greater the impact speed, the higher the risk level of the TAR incident [8]. Viano et al. discovered that TAR was associated with intense compression or direct chest impact. The ruptures of the aorta have been often transverse to the vessel long axis or circumferential [9]. Strassmann found that these ruptures often comprise the media and the intima [10].

Numata et al. analyzed the blood flow of the aortic arch using CFD. To get analytical data concerning aortic disease, they assessed the effect of thoracic aortic aneurysms on blood flow inside the aortic arch. They found that a dilated aorta creates a turbulent flow pattern in the aortic arch. Furthermore, with 75% of the flow from the right subclavian arterial, blood flow from the heart reached the subclavian artery and left common carotid only for a short period throughout the peak of systole [11].

**Figure 1:**
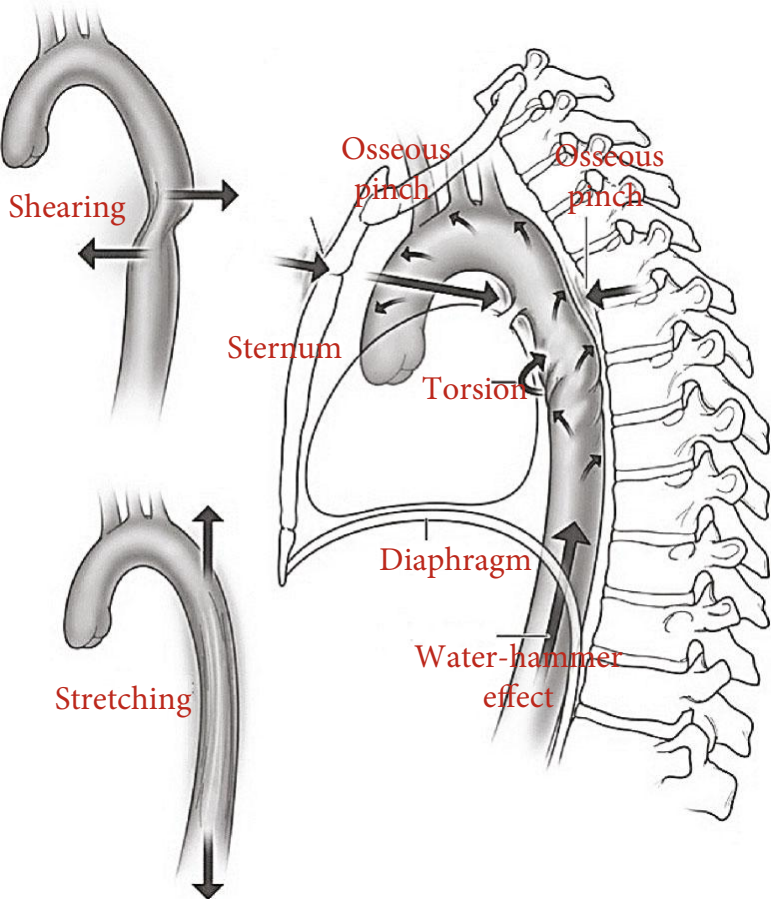
Various proposed mechanisms of TAR [1].

Despite the instant lethality of this very serious injury, there is still uncertainty regarding its mechanistic pathogenesis; with deceleration, osseous pinch, torsion, longitudinal stretch, and water-hammer effects being cited as the causative mechanisms.  Figure 1 shows the various proposed mechanisms of TAR.

The most commonly accepted theory proposes a combination of rapid deceleration with severe chest compression with hyperflexion of the spine and traction at the isthmus leads to TAR [12–18]. The other concept is a shoveling effect as a lower thoracic impact results in an upward movement of the mediastinum causing isthmus severe torsion [18]. The “osseous pinch” model recommends that the proximal descending aorta is compressed amid the sternum, upper thoracic cage, and anteriorly with the vertebral column posteriorly [19]. Another theory suggests the water-hammer effect, whereby sudden occlusion at the diaphragmatic outlet causes an acute rise in aortic pressure disrupting the aortic isthmus [16].

Keeping in view this uncertainty, the injury mechanism of TAR must be studied in detail to optimize protection design and thus decrease the risk of TAR in car crashes. Instead of real dummy impact tests, some researchers have made several body finite element models to make the simulation accurate and reduce experiment costs. However, due to faulty modeling and simulation techniques, there are still improvements that can be made, since the model can include more details in order to confirm the mechanism of TAR.

### 1.2. Development of Dummy Modeling

There have been several human thorax FE models introduced by different researchers. One of the primary finite element models of the human thoracic skeleton was made by Chen and Roberts to examine the biomechanical behavior of the human chest. Later, Feng and Sundaram developed a different 3D model using a beam element approach. In their model, musculature, sternum, and bony ribcage were represented by the membrane, plate, and beam elements [20]. A moreA more accurate human thorax model was developed by Eppinger and Plank [21], whereas a basic model of a human torso was generated by Huang and his coworkers [22] to forecast the parameters of side-impact injury. Utilizing geometrical data from the Schneider team, a FE model of the human thorax for side-impact was created by the Wang team [23, 24] for a seated male (mid-sized) in a driving position. The model contained 11075 shell elements and 4333 solid elements. To survey an extensive range of impact conditions, a complete human model (the number of elements was limited) was created by the Lizee team [25]. A 50^th^ percentile adult male as the total human model was developed for safety by Toyota Central R&D Labs., Inc. [26]. The linear elastic material model was used for the aorta. Moreover, the brachiocephalic trunk, greater vasculature from the aorta, left subclavian artery and left common carotid artery did not result all the way up to the head that has been assumed to make the aortic arch to be stretched during head with neck movement. However, none of the studied models of the human body have had an adequately precise model of the aorta or sufficient anatomical details in the thoracic cavity to forecast TAR [27–31].

To attain an understanding of the mechanisms of potential injury, FE models, and the Lumped parameter of the human thorax have been developed. So far, most studies focus on the relationship between chest deceleration and TAR injury with the isthmic part. No research has focused on studying the interior behavior of the aorta itself and the connections that limit the aorta to move and transfer kinetic energy. In this study, we have used CT scan pictures and reconstructed them to develop the model in MIMICS. FE analysis using ANSYS was employed to explore the kinematics of the aorta. The model was more realistic than other models developed so far, which helped us to obtain further specific information to minimize the risk of aortic injury. The objectives of this study were to improve the finite element model (especially the isthmic part in the aorta) with higher specificity and precision, to simulate the aortic movement after a car crash under different conditions, to obtain more comprehensive data, and to observe the dynamic simulation process of the traumatic rupture of the aorta and analyze the aortic rupture.

### 1.3. Methodology

The use of the FE method to study the dynamic behavior of the human body during a car crash has become a popular and convenient method. It can help researchers obtain exact details about the process of aortic injury and also reduce the high cost of using crash test dummies.

### 1.4. Chest Reconstruction Based on Medical Imaging

Computed tomography (CT) can distinguish different body structures including blood vessels if intravenous contrast materials are used. Observing and analyzing the vascular system through CT and magnetic resonance imaging (MRI) has become effective in studies of hemodynamic research [32, 33].

MIMICS is an interactive modeling software including MIMICS and 3-MATIC, which provide segmentation function and visualization. The latest version of MIMICS (version 19.0) establishes a mature rapid prototyping system. For instance, it provides easy operability, visual rapid modeling, precision modeling, and flexible interface all in one. The software performs segmentation and converts anatomical data from 2D images to a 3D model based on the absorption coefficients of different tissues [34].

**Figure 2:**
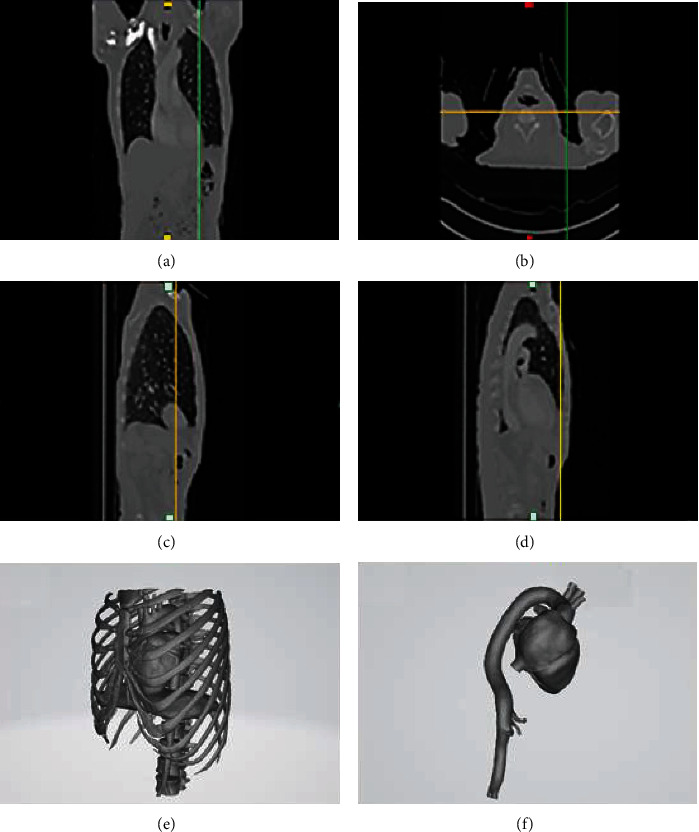
Modeling result of the whole chest in MIMICS (a) front view, (b) top view, (c, d) side views of chest MRI pictures, (e) general view of the whole model, and (f) general view of the aorta-heart model.

In our study, we used 426 CT slices conducted from a CT scanner (Philips Medical from Hamad General Hospital, Doha, Qatar). We selected a visible thresholding range to the filtrate and extracted approximate images of the rib cage, spine, lungs, heart, and aorta separately. After the region growing, the calculation of the 3D model was repeated, and then, the mask in 3D was edited to improve the model. The whole thoracic aorta cavity with right subclavian, left common carotid artery, left subclavian artery, and isthmus area was extracted. The automatic process generated a description of the geometry of organ contour and manual image processing was also necessary to improve the model. After generating the contour line of each slice, the whole surface was created using all the contour lines. Here, we used smooth and triangles reduce to delete insignificant little vessels to simplify the desire models. Finally, we obtained the whole chest model with 1598508 triangles and 799137 points (Figures 2(a)–2(f)). For the aorta and heart part, there were 361034 triangles and 180511 points. Figures 2(a)–2(d) show the modeling result of the whole chest in MIMICS at different views. Figures 2(e) and 2(f) show the general views of the whole and the aorta-heart models.

### 1.5. 3D Model Postprocessing

**Figure 3:**
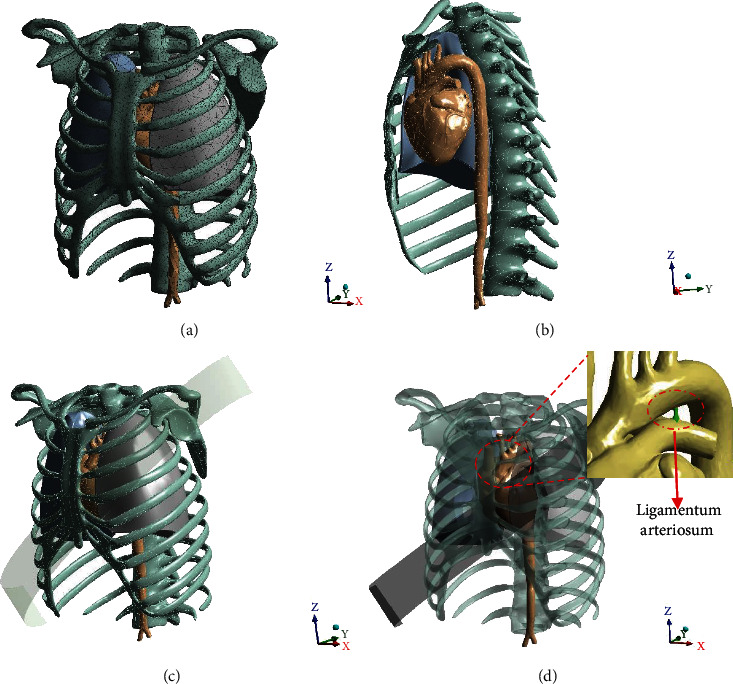
ANSYS 3D views of the whole model (a) with mesh (aorta, heart, lungs, and ribcage), (b) the cross-section, (c) the model with the seat belt, and (d) the transparent model with an emphasis on aorta and heart along with ligamentum arteriosum.

Before outputting the stereolithography (STL) format of the model, 3-Matic was used to optimize the number and shapes of triangles to reduce the time of computation during FE analysis in ANSYS. Meanwhile, a software named GEOMAGIC was also adopted to check and optimize the nodes and elements of the chest model. Finally, we simplified the aorta and heart parts into 4808 nodes and 5304 elements without geometrical changes. The whole simulation model includes aorta, lungs, heart, ribcage, and only aorta.  Figure 3 shows ANSYS 3D views of the whole model with mesh (aorta, heart, lungs, and ribcage), the cross-section, the model with the seat belt, and the transparent model with an emphasis on aorta and heart along with ligamentum arteriosum. Due to interface limits between the MIMICS and the ANSYS, we transferred the format of the model from STL to STP after optimization in the GEOMAGIC.

### 1.6. Process of the Dynamic Simulation

ANSYS WORKBENCH is a highly integrated simulation platform. It provides an intelligible graphical user interface (GUI), and users can create geometries, edit models, and set up boundary conditions in a visible interface conveniently. Due to excellent bidirectional connections to popular CAD software, target models can be converted, read, and modified unimpededly. Furthermore, users can examine the results and correct parameters at any moment during postprocessing based on powerful computation [35–37].

#### 1.6.1. Material Properties

To obtain an accurate dynamic response of the aorta, the material properties need to be considered. Many researchers made efforts to study the precise biomechanical properties of body tissues. The organs' material properties utilized in this work were provided by FE models in the literature.  Table 1 shows the tissue properties used for viscera and bone. Properties such as Poisson ratio, density, and modulus of elasticity were defined in our FE model [38, 39].

#### 1.6.2. Explicit Dynamics Analysis

An explicit dynamics analysis was utilized to determine the dynamic response of a structure due to impact or rapidly changing time-dependent loads. Momentum exchange between moving bodies and inertial effects are usually important aspects of the type of analysis being conducted. This type of analysis can also be used to model mechanical phenomena that are highly nonlinear. Nonlinearities may stem from the materials, (for instance, plastic flows, hyperelasticity, and failure), from contact (for instance, high-speed collisions and impact), and the geometric deformation (for instance, collapse and buckling). Events with time scales of less than 1 second (usually of the order of 1 millisecond) are efficiently simulated with this type of analysis.

#### 1.6.3. Explicit Transient Dynamics

The partial differential equations to be solved in an explicit dynamics analysis express the conservation of mass, momentum, and energy in Lagrangian coordinates. These, together with a material model and a set of initial and boundary conditions, define the complete solution of the problem.

For a contact system, by applying the finite element technique to the variational equation the following equations give rise:

(1) $$\begin{array}{c} m\ddot{d}\left(t\right)+F^{\mathrm{int}}\left(d\left(t\right)\right)+F_{c}\left(d\left(t\right)\right)=F^{\mathrm{ext}}\left(t\right) \end{array}$$

which is to hold for all *t* *ϵ* (0, *T*) and which is subject to initial conditions;

(2) $$\begin{array}{c} d\left(0\right)=d_{0},\dot{d}\left(0\right)=v_{0} \end{array}$$

where *Fint*, *Fc*, and *Fext* are the internal force vector, the contact force vector, and the applied force vector, respectively.

The most common explicit time integrator can be written in the following form for a typical time step Δ*t*;

(3) $$\begin{array}{c} a_{n}=m^{-1}\left(F_{n}^{\mathrm{ext}}-F^{\mathrm{int}}\left(d_{n}\right)-{\left.F_{c}\right|}_{n}\right),\\ V_{n+\frac{1}{2}}=V_{n-\frac{1}{2}}+∆t_{n},\\ d_{n+1}=d_{n}+∆tV_{n+\frac{1}{2}} \end{array}$$

where subscripts *n*, *n* + 1, etc. specify indexing with time, and *V* and *m* are the velocity and mass (a diagonalized mass matrix), respectively [40].

For the Lagrangian formulations currently available in the explicit dynamics system, the mesh moves and distorts with the material it models and conservation of mass is automatically satisfied. The density at any time can be determined from the current volume of the zone and its initial mass as shown in Eq. (4);

(4) $$\begin{array}{c} \frac{\rho_{0}v_{0}}{v}=\frac{m}{v} \end{array}$$

Where *p*, *v*0, *v*, and *m* are density, initial volume, volume, and mass, respectively.

The partial differential equations that express the conservation of momentum (as expressed by Eqs. (5), (6), and (7)) relate the acceleration to the stress tensor *σij*.

(5) $$\begin{array}{c} \rho a_{x}=F_{x}+\frac{\partial \sigma_{xx}}{\partial_{x}}+\frac{\partial \sigma_{xy}}{\partial_{y}}+\frac{\partial \sigma_{xz}}{\partial_{z}} \end{array}$$

(6) $$\begin{array}{c} \rho a_{y}=F_{y}+\frac{\partial \sigma_{yx}}{\partial_{x}}+\frac{\partial \sigma_{yy}}{\partial_{y}}+\frac{\partial \sigma_{yz}}{\partial_{z}} \end{array}$$

(7) $$\begin{array}{c} \rho a_{z}=F_{z}+\frac{\partial \sigma_{zx}}{\partial_{x}}+\frac{\partial \sigma_{zy}}{\partial_{y}}+\frac{\partial \sigma_{zz}}{\partial_{z}} \end{array}$$

Where *σ*, *ax*, *ay*, and *az* are stress and accelerations in *x*, *y*, and *z* directions. Also, *Fx*, *Fy*, and *Fz* are the forces in *x*, *y*, and *z* directions.

Conservation of energy is expressed by Eq. (8):

(8) $$\begin{array}{c} \dot{E}=\frac{1}{\rho }\;\left(\sigma_{xx}{\dot{\varepsilon }}_{xx}+\sigma_{yy}{\dot{\varepsilon }}_{yy}+\sigma_{zz}{\dot{\varepsilon }}_{zz}+2\sigma_{xy}{\dot{\varepsilon }}_{xy}+2\sigma_{yz}{\dot{\varepsilon }}_{yz}+2\sigma_{zx}{\dot{\varepsilon }}_{zx}\right) \end{array}$$

These equations are solved explicitly for each element in the model, based on input values at the end of the previous time step. Small-time increments were utilized to ensure the stability and accuracy of the solution. Note that in explicit dynamics, we do not seek any form of equilibrium; we simply take results from the previous time point to predict results at the next time point. There is no requirement for iteration.

In a well-posed explicit dynamics simulation, energy, momentum, and mass should be conserved. Only mass and momentum conservations are enforced. Energy is accumulated over time and conservation is monitored during the solution. Feedback on the quality of the solution was provided via summaries of momentum and energy conservation (as opposed to convergent tolerances in implicit transient dynamics) [41].

#### 1.6.4. Boundary Conditions

First, we set up a standard global coordinate system and the gravitational field environment. We assumed an idealization movement of aorta during front and side-impact. In ANSYS, we defined the negative *Y*-axis as the front direction and the *X*-axis as the side direction. Subclavian arteries of aorta and carotid arteries limit the movement from the top. And the connection between the vertical part of aorta and spine limits the movement. To obtain a comprehensive movement trend and mechanical analysis, we adopted 4 different initial velocities in *Y*-axis (10, 15, 20, and 25 m/s) direction and 3 different initial velocities (15, 20, and 25 m/s) in *X*-axis direction. Then, the heart was supposed to move forward, and the dynamic behavior of the aorta was observed that happened in the isthmus region. Besides, to include the effect of the blood pressure on the aorta's wall during deceleration, we considered a hydrostatic pressure in the aorta and heart. Here, the shell face of the aorta defined as the side of the shell on which to apply the hydrostatic pressure load. The magnitude and direction of the hydrostatic acceleration were specified. This was a negative acceleration due to the deceleration of aorta during the impact which led blood to undergo deceleration.

#### 1.6.5. Steps of Dynamics Simulation in ANSYS

At first, we imported the whole model into ANSYS. Due to the differences in tissue properties, we established a database that includes all the parameters such as Poisson ratio, density, and modulus of elasticity. Then, we defined the properties of different tissues. Hydrostatic pressure was considered in the aorta to simulate the effect of blood on the aorta's wall during deceleration. Also, for the dynamic part, we defined the boundary conditions such as standard coordinate system, the initial velocity of the model, and gravitational field fixation defining all contact areas in between aorta, spinal cord, heart, lungs, and ribs to simulate all interactions. After finishing the simulation of all cases, the results were checked in the report preview.

## 2. Results and Discussion

Acceleration is a common substitute index of the severity of impact, but the correlation amid the outcome of injury and severity of impact always varies as soon as diverse modeling techniques are conducted (locating a load to the body). Numerous researches have been carried out to evaluate the reason for the injury of the aorta by segregating other potential injury mechanisms such as distraction of the arch and chest compression [9, 37–39]. Even though the researches indicate that in the absence of other factors, those mechanisms are the reason for injury of the aorta; however, none of those investigations demonstrate that their particular mechanism of injury is the main reason for injury of the aorta in the field.

The preponderance of aortic rupture at the isthmus is currently explained by various possible mechanisms [1, 12–18]: (1) an abrupt stretching by deceleration forces acting upon an inherently weak part of the aortic wall, at the attachment of the arterial ligament. (2) Osseous pinch on the aorta by being compressed between the sternum and the vertebral column. (3) Longitudinal traction, where the neck arteries coming out of the aortic arch vertically pull upon the aortic arch with a shearing force. (4) Water-hammer phenomena whereby there is an abrupt increase in the intravascular pressure. Although, in general, the first theory of deceleration forces acting upon a naturally weak area of the isthmus is favored, there is no conclusive evidence of the root cause mechanism of this instantly fatal injury. It may be that different mechanical factors and mechanisms are cocontributors in the genesis of TAR [42].

**Figure 4:**
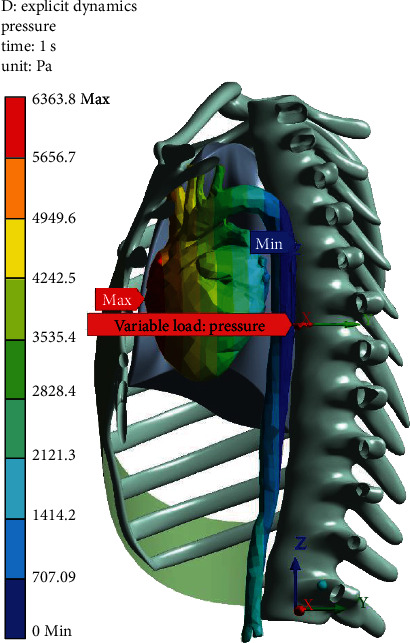
Hydrostatic pressure simulation.

To simulate the effect of blood pressure on the aorta during deceleration, the hydrostatic pressure was applied to the heart-aorta model (Figure 4). The hydrostatic pressure was 6363.6 Pa at the heart, 2828.4 Pa at the aortic arch, and 707 Pa at the abdominal aorta.

**Figure 5:**
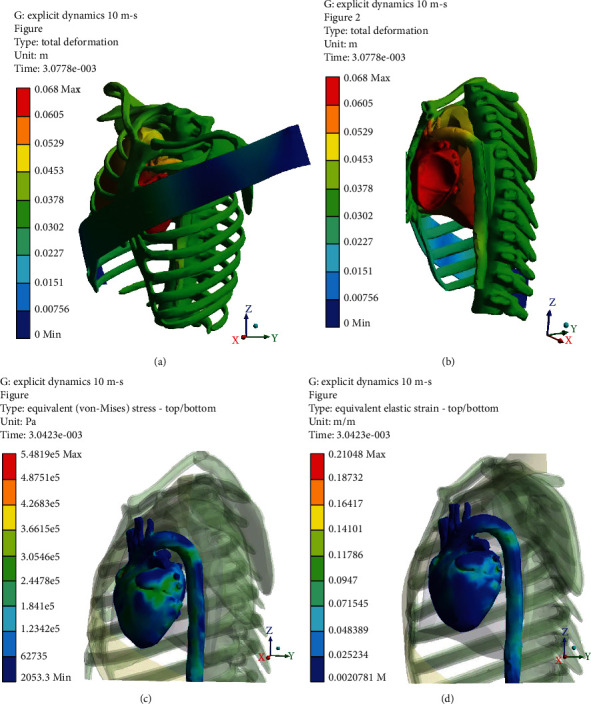
*Y*-axis impact at 10 m/s (a) total deformation (front view), (b) cross-section (side view), (c) equivalent stress, and (d) equivalent elastic strain.

**Figure 6:**
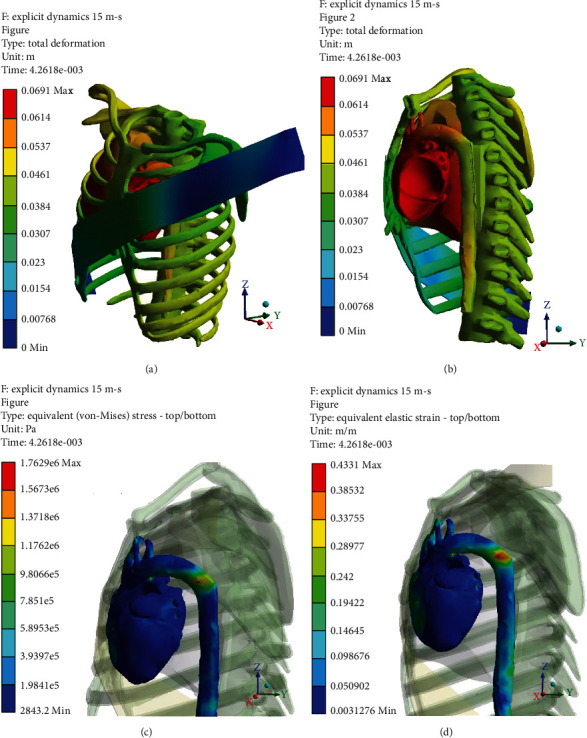
*Y*-axis impact at 15 m/s (a) total deformation (front view), (b) cross-section (side view), (c) equivalent stress, and (d) equivalent elastic strain.

**Figure 7:**
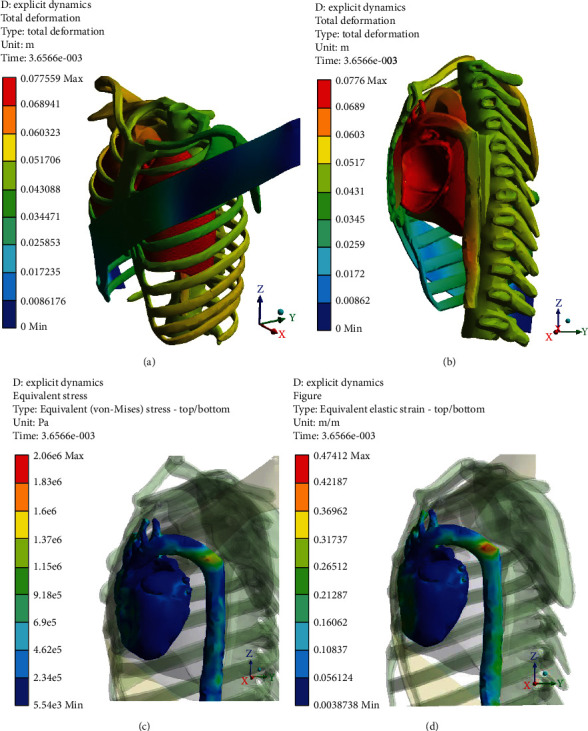
*Y*-axis impact at 20 m/s (a) total deformation (front view), (b) cross-section (side view), (c) equivalent stress, and (d) equivalent elastic strain.

**Figure 8:**
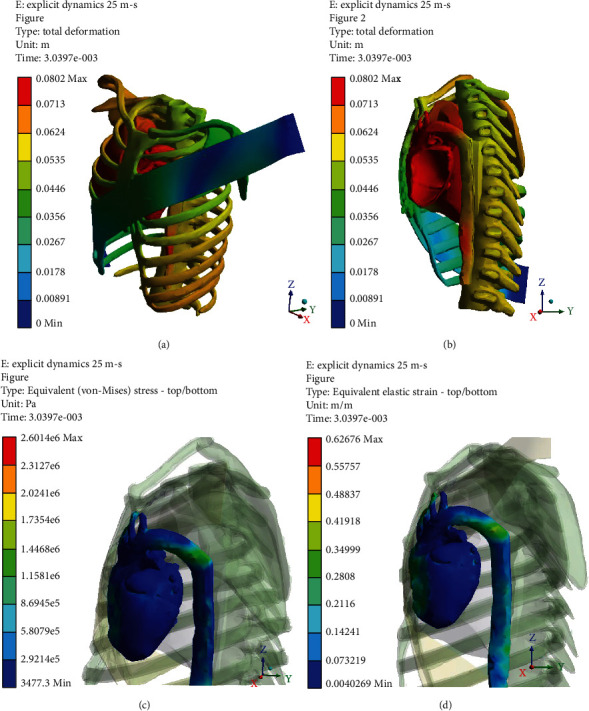
*Y*-axis impact at 25 m/s (a) total deformation (front view), (b) cross-section (side view), (c) equivalent stress, and (d) equivalent elastic strain.

**Figure 9:**
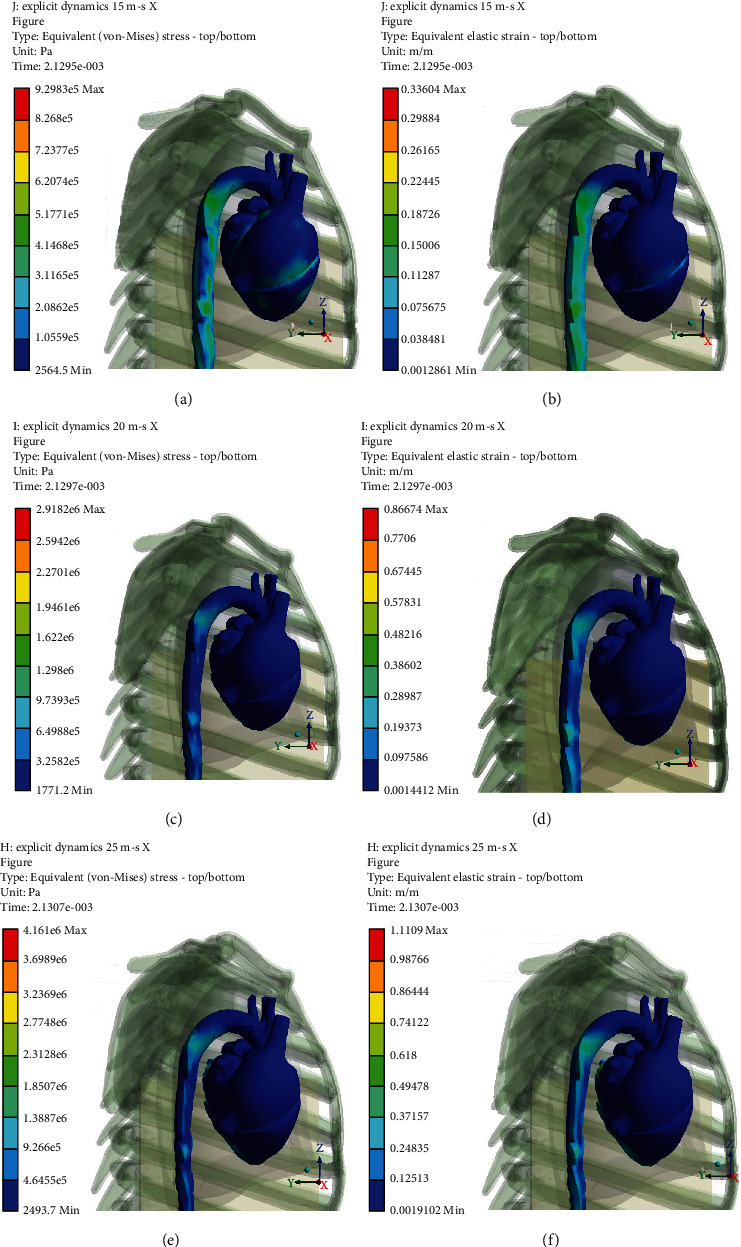
Equivalent stress and equivalent elastic strain for impact at *X*-axis under different velocities.

Figures 5–8 (e and f) show different von Mises stresses and strain FE analyses predicted by the model under different initial velocities. As can be seen, the isthmus area of the aorta and at the pulmonary aorta junction stresses and strains are considerably high compared with the other regions. For the front movement case (*Y*-axis), the maximum stress and strain (5.4819e6 Pa and 0.21048) concentrate on the connection between the aortic arch and the pulmonary artery. When the velocity increases from 10 to 25 m/s, the maximum stress and strain are yet in the same region, but the average stress and strain of the surroundings increases from 3.0546e5 Pa and 0.11786 to 1.4468e6 Pa and 0.34999, respectively. When the velocity increases to 20 m/s, the bending and deformation at the region between the aortic arch and the pulmonary artery can be observed clearly, and the maximum stress and strain increased to 2.06e6 Pa and 0.47412, respectively. When increasing the velocity to 25 m/s, the maximum stress and strain reached 2.614e6 Pa and 0.62676, respectively, and concentrates on the isthmus region. Furthermore, the equivalent stress and equivalent elastic strain for the impact in the *X*-axis under different velocities are shown in Figures 9(a)–9(f). The stress and strain at the connection between the aortic arch and the pulmonary artery for 15, 20, and 25 m/s velocities are 5.1771e5, 0.22445, 1.622e6, 0.57831, 2.3128e6, and 0.618, respectively. When the velocity increases, the heart moves sideways and generates a stretch. The results of several simulations show that the maximum aortic stresses were located at the outer area to the left subclavian artery, in the peri-isthmic area. It was found that the maximum stress region concentrates on the isthmus part with stretching in the aortic arch, and it extends to the upper heart part with an increase in the velocity in the *X*-axis [27–29]. For the *Y*-axis, the average stress and strain concentrate on the isthmus region but doing a compressional movement that extends to the aortic arch. However, under the same velocity conditions, the stresses and strains at the aortic arch for the side direction (*X*-axis) impact are all less than the front direction crash for the corresponding velocities.

Although the FE analysis results show that the isthmus region of the aorta is under high stress, the way that the applied forces to the thorax for a different range of impacts are transferred to the thoracic cavity to generate damage to the aortic isthmus is complicated. An abrupt elongation of the aorta is acknowledged by the fundamental mechanism to cause TAR. The aorta's isthmus is placed at the intersection amid the fixed and mobile parts of the aorta. The fourth and the underlying intercostal pedicles confine the descending aorta beneath the isthmus. The dislocation of the aorta's upper moveable parts and the heart filled with blood in the pericardiac cavity in a caudal direction place the isthmus section under tension, resulting in eventually tearing. It can be indicated from the FE analysis that the bending generated at the aortic arch region associated with the stretching, occurred at the ascending aorta region and is the leading factor of the aorta's injury.

The deformation values predicted by finite element analysis within the peri-isthmic area for the *Y*-axis impact for velocities 10, 15, 20, and 25 m/s were 0.0605, 0.0614, 0.068, and 0.0713 m, respectively. The finite element analysis shows that the average true stresses for velocities range 10-25 m/s for the *Y*-direction ranging from 3.0546e5 to 1.4468e6 Pa and velocity range 15-25 m/s for the *X*-direction ranging from 5.1771e5 to 2.318e6 Pa show close agreement with the 1.05 to 4.15 MPa range of failure in the study (biaxial tissue tests for the peri-isthmic area) performed by Hardy et al. [42].

The average equivalent elastic strains ranged from 0.2187 to 0.6267 (for 10-25 m/s velocity ranges) in the *Y*-axis and from 0.18725 to 0.6180 (for 15-25 m/s velocity ranges) in the *X*-axis show very close agreement with the range of failure inferred from tissue tests for the peri-isthmic area (0.068-0.546) provided by Shah [43].

## 3. Conclusion

The objective of this work was to observe the inner behavior of aorta during a car crash and analyze the major factors leading to TAR. To have an accurate model, it is necessary to focus on the inner mechanism of the traumatic rupture of the aorta. Here, we used an advanced 3D reconstruction software MIMICS based on high-definition CT pictures. The model was then imported into simulation software ANSYS. Hydrostatic pressure was applied to the heart and the aorta to simulate the effect of the blood's weight on the aorta's wall during the deceleration. The hydrostatic pressure was 6363.6 Pa at the heart, 2828.4 Pa at the aortic arch, and 707 Pa at the abdominal aorta. Next, we analyzed the front and the side movement of the whole chest model during the car crash simulation with a different velocity ranging from 10 m/s to 25 m/s. The results show that when the velocity increases from 10 to 25 m/s in the *Y*-direction crash, the max stress and strain varied from 5.4819e5 to 2.614e6 Pa and 0.21048 to 0.62776, respectively. With increasing in velocity from 15-25 m/s in *X*-direction crash the stress and strain at the aorta arch increased from 5.17771e5 to 2.3128e6 Pa and 0.22445 to 0.618, respectively. Among all the cases, the max stresses all occurred in the isthmus region for the *Y*-direction crash. Finally, it can be concluded that the bending generated at the aortic arch is a leading factor in TAR compared to the stretching that occurs at the ascending aorta.

## Figures and Tables

**Table 1:** Material Properties of organs.

Tissue	Material model	Poisson ratio	Density (gram/mm^3^)	Young's Modulus (MPa)
Heart	Elastic	0.45	0.001	0.5
Lung	Elastic	0.45	0.001	0.5
Aorta	Elastic	0.3	0.001	25
Cortical bone	Elastic-plastic	0.3	0.006	14000
Blood	Liquid	Coefficient of viscosity (kg.s/m) = 3.5 × 10^−3^	0.00105	—
Spongy bone	Elastic	0.4	0.001	50

## Data Availability

This manuscript is part of a thesis for a student who is the first author in this paper. The thesis is published at Hanyang University Library (https://lib.hanyang.ac.kr/#/search/detail/17598775).
